# The Psychometric Properties of the Center for Epidemiological Studies Depression Scale (CES-D) for Iranian Cancer Patients

**DOI:** 10.31557/APJCP.2019.20.9.2803

**Published:** 2019

**Authors:** Hamid Sharif Nia, Maryam Rezapour, Kelly A. Allen, Saeed Pahlevan Sharif, Azar Jafari, Hojjat Torkmandi, Amir Hossein Goudarzian

**Affiliations:** 1 *School of Nursing and Midwifery Amol, *; 2 *Psychiatry and Behavioral Sciences Research Center, Addiction Institute, Department of Psychiatry, *; 5 *Student Research Committee, Mazandaran University of Medical Sciences, Sari,*; 6 *Operating Room Group, Department of Nursing, School of Nursing and Midwifery, Zanjan University of Medical Sciences, Zanjan, Iran, *; 3 *The Melbourne Graduate School of Education, the University of Melbourne, Melbourne, Australia,*; 4 *Taylor’s Business School, Taylor’s University Malaysia, Subang Jaya, Malaysia. *

**Keywords:** Cancer, depression, epidemiological, psychometrics, Iran

## Abstract

**Objectives:**

The Center for Epidemiologic Studies Depression Scale (CES-D) was specifically created to assess depression in cancer patients. However, to date, the CES-D has not been validated in Farsi. Therefore, this study aimed to assess the psychometric properties of the CES-D in Iranian cancer patients.

**Methods:**

During a three-month period (October to December, 2015), a total of 380 cancer patients completed a Farsi version of the CES-D. The construct validity of the scale was evaluated by exploratory factor analysis. Reliability was assessed using Cronbach’s alpha and McDonald Omega. All of the statistical procedure were run by SPSS 22 (SPSS Inc., Chicago, IL, USA).

**Results:**

The construct validity of the CES-D determined three factors (somatic affect, negative affect, and positive affect), which explained 65.60% of the total variance. The internal consistency was greater than 0.70.

**Conclusion:**

Findings revealed that the Farsi version of the CES-D has acceptable validity and reliability, which can be used to measure depression in Iranian cancer patients.

## Introduction

Cancer is the second most common cause of death in developed and industrialized countries (Ferlay et al., 2015). In 2017, it was estimated that approximately 100 million people had some form of cancer globally that claimed an estimated 1.9 million lives (Institute for Health Metrics and Evaluation, 2017). It has been reported that cancer rates have increased by up to 68% since the 1990s and this prevalence is predicted to rise (Edwards et al., 2010). In Iran, the prevalence of cancer increased by 19% between 2008 and 2012 (Mohebbi et al., 2017). It is predicted that the rates of cancer will multiply in Iran by 2035 (Mohebbi et al., 2017).

Cancer patients can face negative psychological sequelae that can accompany their illness and treatment (Hong and Tian, 2014). Depression is a commonly occurring psychological disorder in patients with cancer which can present as both debilitating and chronic (Hong and Tian, 2014; Aghakhani et al., 2011). Globally, approximately 33% of cancer patients around the world are suffering from depression, and this trend is also observed in Iran (Arrieta et al., 2013; Jasemi et al., 2016). 

Depression has been found to be associated with quality of life (Higginson and Costantini, 2008), increased risk of suicide (Couper et al., 2013), exacerbated suffering (McFarland and Holland, 2018), emotional difficulties and the need for support in family members (Rhondali et al., 2015), increased time in hospital (Smith, 2015), and increased risk of mortality (due to the negative effects during treatment) (Chida et al., 2008; Lutgendorf et al., 2010; Satin et al., 2009). 

Notably, the religious and cultural context in which a person lives plays a central role in understanding how people perceive, manage, and respond to depression (Kleinman and Good, 1986). The religious and cultural backgrounds of people have been found to influence a person’s experience depression (Sharif Nia et al., 2017). For example, it is widely established that people from Eastern contexts are more likely to emphasize physical effects of depression, whereas people from Western cultures are more likely to emphasize the psychological effects (Ryder et al., 2008). The cultural context for cancer patients may also influence the perceived implications of depression (Davies, 2016). For example, Islamic populations may view depression differently from other cultures where beliefs about death and the afterlife can vary (Sharif Nia et al., 2017). Cultural context is an important consideration in assessing depression, thus it is necessary to develop an accurate tool for Iranian cancer patients and not assume that tools developed in other contexts are appropriate (Gilbert, 2016). 

While various measures of depression exist [e.g., Hospital Anxiety and Depression Scale (HADS) (Janda et al., 2017); Beck Depression Inventory (BDI) (Shinn et al., 2017); and Center for Epidemiologic Studies Depression Scale (CES-D) (Siddaway et al., 2017)], the CES-D has been evaluated and used with various populations including patients with cancer (Chen et al., 2010; Crespi et al., 2008; Den Oudsten et al., 2009; Santor et al., 2006). 

The CES-D was initially developed and evaluated by Radloff (1977) to study the epidemiology of depressive symptoms in the general population. The scale combined items from previously validated scales, including the Beck Depression Inventory, the Zung Self-Rating Depression Scale, and the Raskin Depression Rating Scale. The CES-D was designed to capture four factors: depressed affect (5 items), anhedonia (4 items), somatic complaints (6 items), and interpersonal concerns (2 items). It has been adapted and validated in different languages, including Chinese (Wang et al., 2013; Yang et al., 2015; Zhang et al., 2012); Vietnamese (Thanh et al., 2016); and French (Cartierre et al., 2011) and used as a research tool in longitudinal studies (Bakitas et al., 2009; Ghazali et al., 2014). Based on a review of available literature, a Farsi version of the CES-D has not been created and tested. Therefore, the present study aimed to assess the psychometric properties of the CES-D in Iranian cancer patients.

## Materials and Methods

*Study design and sample*

During a three-month period (October to December, 2015) a total of 600 patients were admitted to the oncology ward of a university hospital in Sari, Iran. Participants of the study had (i) a current diagnosis of cancer; (ii) were aged 18 years or older; (iii) had not taken antidepressants within the last 6 months; (iv) had the ability to read and write Farsi; (v) demonstrated the capacity to be alert, oriented, and cognitively intact; (vi) had been hospitalized for at least 24 hours or more; (vii) were not being transferred to another hospital; (viii) had no other critical co-occurring conditions (e.g., alcohol or other drug addiction); and (ix) displayed no linguistic and/or physical problems that impeded their abilities to participate in the current study. 

The minimum sample size for conducting a factor analysis was determined to be at least 5 to 10 times the number of tested items (Plichta et al., 2013). Out of the 600 participants, 150 patients were excluded based on the inclusion criteria stated above. A total of 52 participants were under the age of 18, 71 had taken antidepressants during the last 6 months, and 27 had acute medical conditions. Of the remaining 450 eligible participants, 380 agreed to participate, and they had a response rate of 63.3 %.

*Data collection and procedures*

Participants were allocated to the study using non-random sampling (i.e., accessible sampling). That is, patients who met the inclusion criteria and had been referred by nursing staff. Informed consent was obtained after participants were informed about the purpose of the study and their right to withdraw at any time. Approximately half of the participants filled out the questionnaire with assistance of an interviewer due to poor eyesight. On average, it took 15 minutes to fill out the self-administered questionnaire.

*The CES-D*

The CES-D is used to measure symptoms associated with depression that have been experienced within one week (Vilagut et al., 2016). Each of the 20 items available in this instrument are measured using the Likert scale in the following way: 0 = rarely or never (less than one day); 1 = occasionally or in few cases (1 to 2 days); 2 = occasionally or a moderate amount of time (3 to 4 days); and 3 = most of the time or all the time (5 to 7 days) (Wang et al., 2013). The total scores range from 0 to 60. Higher scores indicate greater depressive symptomatology (Maass et al., 2015). The validity and reliability of this scale were proven in various studies (Wang et al., 2013; Yang et al., 2015; Zhang et al., 2012).

*Ethical considerations*

This study was approved by the Ethics Committee of Mazandaran University of medical sciences (IR.MAZUMS.REC.95.S.121). Only patients who had agreed and signed the informed consent form participated in this study.

*The Verification of the Farsi Version of the CES-D*

For the present study, the verification procedure included a Farsi translation, synthesis, back translation, and expert committee review. Two scholars independently translated the CES-D from English to Farsi. One translator was a professor from a well-known graduate school of nursing, and the other was an associate professor of a graduate institute of long-term care. Both scholars received doctoral degrees from English-speaking countries. During the translation, they recorded comments and ideas when they encountered controversial phrases or uncertainties about wording, and they made annotations accordingly. Two written reports were then completed (Sperber, 2004).

*Synthesis*

The two Farsi versions were synthesized into one. The two translators deliberated over each word and item and provided their consent to the final version. The corresponding author of this study integrated the translation after careful consideration, then completed a written report (Sperber, 2004).

*Back translation*

Two bilingual scholars without medical backgrounds (Dunbar et al., 2000; Sperber, 2004) translated the synthesized Farsi version back into English. One translator had obtained a degree in linguistics in Malaysia, and the other was a Singaporean graduate student. The main purpose of this endeavor was to determine whether the back-translated version and the synthesized Farsi version were the same in terms of the linguistic content and meaning.

*Construct validity assessment*

To assess the construct validity, the factor structure of the Farsi version of the CES-D was examined by conducting a maximum likelihood exploratory factor analysis (MLEFA), followed by a Promax rotation with SPSS 22 (SPSS Inc., Chicago, IL, USA). Next, 380 patients were asked to complete the Farsi version of the CES-D. The Kaiser-Meyer-Olkin (KMO) test and Bartlett’s test of sphericity were used to check the appropriateness of the study sample and the factor analysis model. The number of latent factors was estimated by using parallel analysis. The items with absolute loading values of 0.3 or greater were regarded as appropriate (Saggino and Kline, 1996).

*Reliability assessment*

The reliability of the Farsi version of the CES-D was first assessed by evaluating its internal consistency and calculating the Cronbach’s alpha (α) and McDonald’s omega (Ω) (Javali and Gudaganavar, 2011). A reliability of 0.7 or greater showed a satisfactory internal consistency (Jorritsma et al., 2012). Intra-class correlation coefficients (ICC) were used to establish the test–retest reliability of the CES-D over an interval of two weeks by using a two-way mixed ICC that showed an absolute agreement at the level of individual items. The results were interpreted as follows: 0–0.2 = low; 0.21–0.40 = fair; 0.41–0.60 = moderate; 0.61–0.80 = substantial; and 0.81–1 = almost perfect (Landis and Koch, 1977). 

*Multivariate normality and outliers*

Univariate distributions were examined for outliers, skewness, and kurtosis. Multivariate distributions were evaluated for normality and multivariate outliers (Sharif Nia et al., 2017). Multivariate normality can be evaluated through the use of the Mardia’s coefficient of multivariate kurtosis. One indication of deviation from normal distribution was a Mardia’s coefficient greater than 8 (Raoprasert and Islam, 2010). Multivariate outliers were evaluated through the evaluation of a Mahalanobis distance (Harrington, 2008). Items with a Mahalanobis distance of p < .001 were considered to be multivariate outliers (Tabachnick and Fidell, 2013).

## Results

*Preliminary analyses and descriptive information*

A demographic profile of 380 cancer patients is summarized in Table 1. Generally, male patients (48.39 ± 13 ± 39; 95% CI: 46.41-50.38) were older than the females (45.33 ± 18.44; 95% CI: 42.79-47.87).

*Construct Validity*

The KMO was 0.911, and the Bartlett’s test of sphericity was significant (P < 0.001), which indicated that the sampling was adequate. Table 2 shows the results of MLEFA on the Farsi Version of the CES-D. The MLEFA revealed that three combined factors accounted for 65.60 % of the variance. Factor 1 (somatic affect) had 7 items. Factor 2 (negative affect) had 5 items, and Factor 3 (positive affect) had 4 items. 

**Table 1:** Demographic Profile of Participants

Characteristic	N (%)	Characteristic	N (%)
Sex		Family history of cancer	
Male	175 (46.1)	Yes	112 (29.5)
Female	205 (53.9)	No	268 (70.5)
Economic status		Depression	
Weak	110 (28.9)	Down	261 (68.7)
Average	204 (53.7)	Up	119 (31.3)
Good	66 (17.4)	Past medical history*	
Education		Cardiac diseases	146 (38.42)
Illiterate	210 (55.3)	Respiratory diseases	57 (15)
Diploma	138 (36.3)	Gastric diseases	141 (37.1)
BS	22 (5.8)	Urinary diseases	36 (9.48)
MSs and above	10 (2.6)	History of cigarette smoking	
Marital status		Yes	71 (18.7)
Single	51 (13.4)	No	309 (81.3)
Married	329 (86.6)	Characteristic	Mean (SD)
Cancer stage		Age	46.74 (16.328)
One	132 (34.7)		
Two	133 (35)		
Tree	92 (24.2)		
Four	23 (6.1)		

*Number of patients who had these diseases

**Table 2:** Factor Analysis for the Persian Version of CES-D in Patients with Cancer

Factors	Factors name	Items	Loading	h^2^	% of Variance	Eigenvalues	Internal consistency
1	Somatic	Q1. I was bothered by things that usually don't bother me.	0.922	0.803	32.121	5.622	α=.916, Ω=.953
		Q2. I did not feel like eating: my appetite was poor.	0.875	0.755			
		Q3. I felt that everything I did was an effort.	0.801	0.721			
		Q11. My sleep was restless.	0.786	0.533			
		Q20. I could not get “going.”	0.762	0.672			
		Q5. I had trouble keeping my mind on what I was doing.	0.681	0.597			
		Q13. I talked less than usual.	0.597	0.642			
2	Negative affect	Q12. I felt that I could not shake off the blues, even with the help from family or friends.	0.824	0.592	18.765	3.620	α=.891, Ω=.885
		Q6. I felt depressed.	0.722	0.634			
		Q14. I felt lonely.	0.691	0.533			
		Q17. I had crying spells.	0.655	0.683			
		Q18. I felt sad.	0.583	0.423			
3	Positive affect	Q4. I felt that I was just as good as other people.	0.819	0.637	14.715	2.510	α=.822, Ω=.854
		Q8. I felt hopeful about the future.	0.726	0.544			
		Q12. I was happy.	0.654	0.613			
		Q16. I enjoyed life.	0.598	0.413			

Abbreviation, h^2^, Communalities

*Reliability*

As reported in Table 2, the Cronbach’s alpha and McDonald’s omega demonstrated good reliability and internal consistency for three factors. The average ICC was 0.841 with a 95% confidence interval from 0.703 to 0.901 (p < .001).

## Discussion

The results of the present study supported a three-factor structure: somatic symptoms, negative affect, and positive affect for the CES-D scale. These three factors explained 65.60% of the variance. 

Thanh et al. (2016) reported that the CES-D scale consisted of two factors (negative affect and positive affect). A three-factor structure of the CES-D has been reported previously in a sample of Arab females (interpersonal problems, somatic symptoms, and positive affect) (Ghubash et al., 2000). Zhang et al., (2012) also presented a three factor model: positive affect, interpersonal problems, and a combination of depressive mood and somatic symptoms of the CES-D scale in a study in rural China. Also, a three-factor structure of the CES-D has been identified by Fountoulakis et al., (2001), which included positive affect, a combination of irritability and problems with interpersonal relationships, and a combination of depressive symptoms and somatic symptoms. 

However, other research has reported four factors. Zhang et al., (2015), Thombs et al., (2008), and Chin et al., (2015) revealed four factors of the CES-D scale (depressed affect, somatic symptoms, positive affect, and interpersonal problems) Hair et al., (2010) stated that in the psychological studies and human sciences, the extraction of factors is appropriate when the explained variance falls between 50% and 60%. 

The present study differs from previous research in terms of the number of factors identified and the participant cohort and context. The three-factor structure of the CES-D was identified in the present study, but a two-factor structure of it (Kwakkenbos et al., 2013; Thanh et al., 2016) and four-factor structure of it (Chin et al., 2015; Thombs et al., 2008; Zhang et al., 2015) were reported in other studies (Ghubash et al., 2000; Zhang et al., 2012).

The first factor identified in our study was somatic affect. Somatic affect can related to fatigue, loss of energy, and physical dysfunctions, such as a disturbance of sleep and appetite (Kapfhammer, 2006). Researchers have found that increased somatic complaints are associated with the onset of depression (Penninx et al., 2013). Also, it seems that individuals with greater somatic affects related depression indicate a reduced awareness of behavioral errors (Bridwell et al., 2015; Northouse et al., 2010). This factor was supported by past research (Chin et al., 2015; Fountoulakis et al., 2001; Ghubash et al., 2000; Thombs et al., 2008; Zhang et al., 2012; Zhang et al., 2015). 

The second factor of the CES-D scale found in the study was negative affect. Negative affect usually refers to feelings of sadness and worthlessness (Olino et al., 2011). Individuals with depressive disorders experience a lower positive affect, higher negative affect, and more self-blame after stressful events (Compton et al., 2013). This factor was also identified in the study of Thanh et al., (2016) and Kwakkenbos et al., (2013).

The third factor identified in the present study was positive affect. This affect plays an important adaptive role in physical and psychological health, and it can prevent depression as a protective factor (Jaser et al., 2011). Positive affect is associated with adaptability and flexible thinking (Danhauer et al., 2013). On the other hand, a reduced positive affect can predict the onset of depression (Nelis et al., 2015). In line with this study, this factor appeared in several other studies as an important aspect of the CES-D instrument (Chin et al., 2015; Fountoulakis et al., 2001; Ghubash et al., 2000; Thombs et al., 2008; Zhang et al., 2012).

According to the findings of this study, the coefficients of internal consistency for the overall CES-D scale indicated that this scale had an acceptable reliability. Also, the reliability of this scale has been assessed by Cronbach’s alpha in several other studies (Lehmann et al., 2011; Makambi et al., 2009; Zhang et al., 2015). For instance, the internal consistency (Cronbach’s α) of the CES-D scale was 0.85, with a test-retest correlation coefficient (r) of 0.64 in the study conducted by Zhang et al., (2015). Also, Makambi et al., (2009) tested the internal consistency of data by Cronbach’s α coefficient and split-half coefficient, which yielded a magnitude of over 0.85 for both indices.

## Limitations

The researchers of this study have ensured that the forward-backward translation method was performed at a high standard, and the original author of the scale confirmed the accuracy of the translation. Apart from this confirmation, there’s always the potential of using a scale that was originally designed for a different population. Cultural differences and language nuances may not be translatable, and test users would be advised to remain cognizant about this potential issue. Also, when the researchers of this study completed questionnaires for patients with vision difficulties, they could have reported biased responses.

## Recommendations

Future validation studies with samples from different populations (as well as longitudinal designs) are suggested to verify the findings of this study. Also, since Iranian populations reside all over the world, it could beneficial to test the tool in Iranians in Europe, Asia, Australia, and the USA in order to determine its generalizability for all Iranian populations.

## Nursing Implication

The Farsi Version of the CES-D can be used by nurses and other hospital staff members in cancer units to screen for symptoms of depression. Also, it can be used in nursing education as a component of the nursing process (including diagnosis). Finally, using a valid, reliable tool in nursing research can demonstrate valuable results that researchers can use in cancer patients. 

In conclusion, this study confirmed acceptable psychometric properties, as well as the factor structure of the CES-D in an Iranian sample. Given these findings, the scale can be used as a valid, reliable tool for the assessment of depression that is experienced by Iranian cancer patients.
